# Supplementary material for: Causal Relationship between Aging and Anorexia Nervosa: A White-Matter-Microstructure-Mediated Mendelian Randomization Analysis
Source: Biomedicines. 2024 Aug 16;12(8):1874. doi: 10.3390/biomedicines12081874 (PMC11351342; doi:10.3390/biomedicines12081874)
Supplement: Supplementary file 1 [file biomedicines-12-01874-s001.zip › Supplementary Figure/Figure S4/Singe SNP plot of MR analysis for TL to ubm-b-1549.pdf]

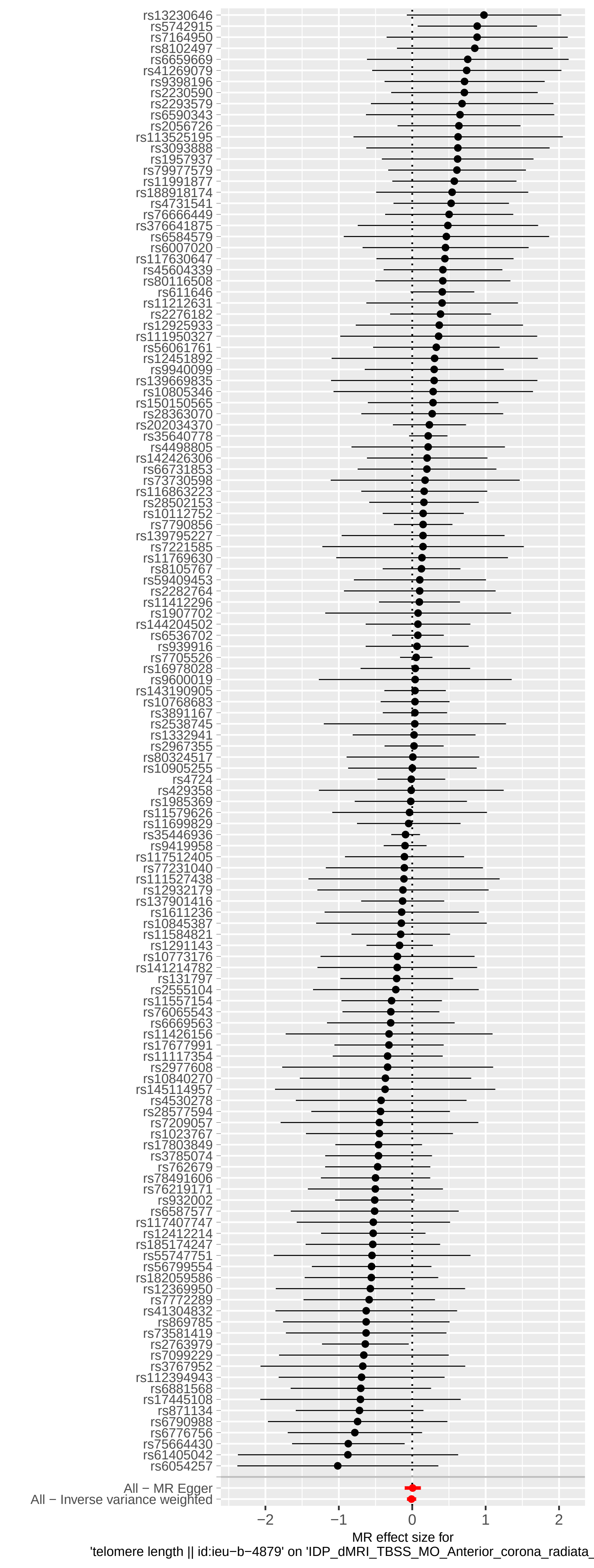

rs13230646
rs5742915
rs7164950
rs8102497
rs6659669
rs41269079
rs9398196
rs2230590
rs2293579
rs6590343
rs2056726
rs113525195
rs3093888
rs1957937
rs79977579
rs11991877
rs188918174
rs4731541
rs76666449
rs376641875
rs6584579
rs6007020
rs117630647
rs45604339
rs80116508
rs611646
rs11212631
rs2276182
rs12925933
rs111950327
rs56061761
rs12451892
rs9940099
rs139669835
rs10805346
rs150150565
rs28363070
rs202034370
rs35640778
rs4498805
rs142426306
rs66731853
rs73730598
rs116863223
rs28502153
rs10112752
rs7790856
rs139795227
rs7221585
rs11769630
rs8105767
rs59409453
rs2282764
rs11412296
rs1907702
rs144204502
rs6536702
rs939916
rs7705526
rs16978028
rs9600019
rs143190905
rs10768683
rs3891167
rs2538745
rs1332941
rs2967355
rs80324517
rs10905255
rs4724
rs429358
rs1985369
rs11579626
rs11699829
rs35446936
rs9419958
rs117512405
rs77231040
rs111527438
rs12932179
rs137901416
rs1611236
rs10845387
rs11584821
rs1291143
rs10773176
rs141214782
rs131797
rs2555104
rs11557154
rs76065543
rs6669563
rs11426156
rs17677991
rs11117354
rs2977608
rs10840270
rs145114957
rs4530278
rs28577594
rs7209057
rs1023767
rs17803849
rs3785074
rs762679
rs78491606
rs76219171
rs932002
rs6587577
rs117407747
rs12412214
rs185174247
rs55747751
rs56799554
rs182059586
rs12369950
rs7772289
rs41304832
rs869785
rs73581419
rs2763979
rs7099229
rs3767952
rs112394943
rs6881568
rs17445108
rs871134
rs6790988
rs6776756
rs75664430
rs61405042
rs6054257
All − MR Egger
All − Inverse variance weighted
−2
−1
0
1
2
MR effect size for
'telomere length || id:ieu-b-4879' on 'IDP_dMRI_TBSS_MO_Anterior_corona_radiata_
